# Relationship between BMI and alcohol consumption levels in decision making

**DOI:** 10.1038/s41366-021-00919-x

**Published:** 2021-08-06

**Authors:** Khushbu Agarwal, Sukru Baris Demiral, Peter Manza, Nora D. Volkow, Paule Valery Joseph

**Affiliations:** 1 National Institute on Alcohol Abuse and Alcoholism, Bethesda, MD USA; 2 National Institute of Nursing Research, Bethesda, MD USA; 3 National Institute on Drug Abuse, Baltimore, MD USA

**Keywords:** Diseases of the nervous system, Cognitive control

## Abstract

**Background:**

Decision-making deficits in obesity and alcohol use disorder (AUD) may contribute to the choice of immediate rewards despite their long-term deleterious consequences.

**Methods:**

Gambling task functional MRI in Human connectome project (HCP) dataset was used to investigate neural activation differences associated with reward or punishment (a key component of decision-making behavior) in 418 individuals with obesity (high BMI) and without obesity (lean BMI) and either at high (HR) or low (LR) risk of AUD based on their alcohol drinking levels.

**Results:**

Interaction between BMI and alcohol drinking was seen in regions of the default mode network (DMN) and those implicated in self-related processing, memory, and salience attribution. ObesityHR relative to obesityLR also recruited DMN along with primary motor and regions implicated in inattention, negative perception, and uncertain choices, which might facilitate impulsive choices in obesityHR. Furthermore, obesityHR compared to leanHR/leanLR also demonstrated heightened activation in DMN and regions implicated in uncertain decisions.

**Conclusions:**

These results suggest that BMI is an independent variable from that of alcohol drinking levels in neural processing of gambling tasks. Moreover, leanLR relative to leanHR, showed increased activation in motor regions [precentral and superior frontal gyrus] suggestive of worse executive function from excessive alcohol use. Delayed discounting measures failed to distinguish between obesity and high alcohol drinking levels, which as for gambling task results suggests independent negative effects of obesity and chronic alcohol drinking on decision-making. These findings highlight distinct associations of obesity and high-risk alcohol drinking with two key constituents of decision-making behavior.

## Background

Heavy drinking is associated with a greater waist-hip ratio in mid-life even when taking other influences into account such as having overweight parents, maternal smoking in pregnancy, and physical inactivity [1, 2]. Further, regular and/or heavy episodic drinking in young adults increases the risk of being overweight or obese [3]. On the other hand, some cross-sectional studies have shown an inverse relationship between moderate alcohol consumption and high waist circumference [4] and the prevalence of metabolic syndrome [5]. A systematic review of large cross-sectional and long-term prospective cohort studies found no conclusive evidence for a positive association between alcohol consumption and weight gain [6]. Moderate to hazardous levels of alcohol consumption have been linked with lower BMI in females due to decreased carbohydrate intake from other sources (for example sucrose) [7]. Reduced energy intake from food or non-alcoholic beverages in heavy alcohol drinkers (both males and females) has been reported through the National Health and Nutrition Examination Survey (NHANES) by various groups [8–10]. However, there are inconsistent reports on the effect of alcohol as a major energy source contributing to the BMI of drinkers. Colditz et al. reported an inverse association between alcohol consumption and BMI, particularly in women, which could be related to alcohol calories being less efficiently utilized [7]. In contrast, higher total energy was associated with higher BMI in male heavy drinkers as compared to those consuming lower quantities of alcohol on days when drinking occurred [10]. Furthermore, some epidemiological studies have reported that energy intake from alcohol beverage type and drinking pattern (i.e., high intensity/volume, high frequency) contribute to total energy intake and are associated with excess body weight amongst young adults [3, 11, 12]. Higher consumption of energy-dense alcoholic beverages was associated with lower diet quality scores in males and females [9]. One of the major adverse effects of higher calorie intake among drinkers is the lower nutrient densities of protein, fat, carbohydrate, and some minerals and vitamins [13].

The metabolic imbalance due to obesity is associated with chronic low-grade inflammation due to elevated circulating pro-inflammatory cytokines. This chronic inflammation extends beyond the adipose tissues to the central nervous system (CNS). Ingestion of a high saturated fat diet increases the expression of inflammatory cytokines in the hypothalamus, which presumably are regulated by microglia [14, 15]. The susceptibility of the CNS to inflammation following high-fat diets was revealed by a rodent study that observed gliosis and inflammation in the hypothalamus within 3 days of high-fat diet exposure [16]. Cognitive impairment and brain dysfunction have been reported with obesity-triggered chronic neuroinflammation. Specifically, a preclinical study revealed activation of the IKK/NF-kB pathway (with constitutive activity in the hypothalamus) resulting in excessive release of inflammatory cytokines such as TNF-α and IL-1β during obesity, which reduced neurogenesis, led to cognitive deterioration and degeneration of hypothalamic stem cells [17]. Accumulating evidence therefore suggests that CNS and cognitive function are deleteriously affected by obesity [18, 19].

Overlaps in the pathways that lead to excessive eating (leading to obesity) and alcohol dependence have been studied. Both obesity and alcohol use disorders (AUD) have been linked to the brain’s reward system [20]. Overconsumption can trigger a gradual increase in the reward threshold, requiring more and more palatable high-fat food or alcohol to satisfy cravings [21]. Evidence suggests an imbalance in three neural systems during the development of AUD and obesity (i) a system that promotes habitual behaviors in response to salient rewards, (ii) an interoceptive system that evaluates internal states and affects responses to uncertain risks and rewards, and (iii) an inhibitory control and decision-making system [22]. Decision-making is often assessed using the Iowa Gambling Task (IGT), which requires inhibition of impulsive responses by factoring in uncertainty, reward, and punishment. Interpretation of IGT performance is challenging since several cognitive constructs are assessed simultaneously, including memory, reward sensitivity, and inhibitory control. Nonetheless, decision-making behaviors have been measured with high ecological validity [23, 24] and impairments in decision-making have been repeatedly demonstrated in addictions and eating disorders [25–27].

Neuropsychological studies support the hypothesis of food/alcohol addiction-related alterations in inhibitory control, emotion regulation, and overall executive function for which a core cognitive trait is decision-making [28]. Individuals with obesity prefer immediate rewards despite negative long-term consequences relative to lean BMI controls [29]. Furthermore, when assessed by the IGT, individuals with obesity and AUD present significant decision-making impairments in overall task performance [26, 27, 30, 31]. Moreover, individuals with comorbid gambling disorder and AUD showed an additive effect in choosing greater immediate rewards reflecting worse decision-making deficits, relative to those with only one condition [32]. Similarly, there is overlap in neurocognitive disruption between obesity and gambling disorder; in gamblers, obesity is associated with decision-making and sustained attention impairments, along with more significant monetary losses from gambling [33].

The published literature suggests that both individuals with obesity and AUD suffer from decision-making deficits; here, we expand this inquiry to investigate differences in neural activation associated with reward or punishment during the gambling task (a key component of decision-making behavior) in individuals with and without obesity (lean) who are either at high or low-risk of AUD. We posited that groups with high BMI and at high AUD risk (obesityHR) would show greater activation to rewards in brain regions critical for inhibitory control, uncertainty, and memory function, compared to the obesity low-AUD risk (obesityLR) group, reflecting greater reward sensitivity. It is also expected that individuals with lean BMI and low AUD risk (leanLR) would exhibit lesser reliance on immediate monetary rewards than high-risk AUD groups (obesityHR and leanHR). Therefore, the results would help us understand the effect of BMI and alcohol drinking on decision-making for low and high reinforcing rewards.

## Methods

### Design and participants

For the present study, we obtained permission from the human connectome project (HCP) to use Open and Restricted Access data from the S1200 (final) release of the Young Adult HCP (ages 22–35). Participants reported no significant history of neurological disorder, cardiovascular disease, or Mendelian genetic disease and did not present any MRI contraindications. General HCP information can be found in Van Essen et al. [34]. Participants were recruited in Missouri and Minnesota. All participants gave informed consent, and all aspects of the protocol were approved by the Washington University School of Medicine Institutional Review Board.

### Categorization of participants into groups

From the list of obesity and lean participants for whom the gambling task fMRI data were available, we included 418 subjects [109 with obesity and 309 lean categorized based on SSAGA_BMICat in HCP dataset]. Subjects were sub-categorized based on their risk status for AUD. Accordingly, high-risk (HR) comprised of both binge (BD) and heavy drinkers (HD), while the low-risk (LR) group included individuals who drink less than 4 drinks on a single day and for less than one day per week in the past 12 months. Furthermore, subjects who met DSM IV criteria for alcohol dependence or abuse were excluded from the LR category. Obesity and leanHR group [(ObesityHR, *n* = 24; 66% males); (LeanHR, *n* = 86; 63% males) and LR group [(ObesityLR, *n* = 85; 35% males); (LeanLR, *n* = 223; 30% males)]. More details on subjects selection criteria are given in Supplementary Fig. S1. Participant characteristics are presented in Table 1. Consistent with the design’s intentions, the two groups differed substantially in BMIs (Table 1). Pearson’s Chi-squared test with Yates’ continuity correction was conducted to see if there is a difference in the number of HR and LR individuals in the obesity and lean groups.

**Table 1:** Participant characteristics (*n* = 418).

	Obesity (*n* = 109; mean ± SD)	Lean (*n* = 309; mean ± SD)	ObesityHR (*n* = 24; mean ± SD)	ObesityLR (*n* = 85; mean ± SD)	LeanHR (*n* = 86; mean ± SD)	LeanLR (*n* = 223; mean ± SD)
BMI (kg/m^2^)	34.99 ± 4.01*	22.99 ± 2.51*	33.35 ± 2.92	35.45 ± 4.17	23.46 ± 1.98	22.81 ± 2.66
Height (inches)	67.39 ± 4.19	67.17 ± 3.79	69.50 ± 4.48	66.79 ± 3.93	68.90 ± 3.58	66.50 ± 3.66
Weight (pounds)	226.12 ± 32.99*	148.17 ± 24.23*	229.92 ± 33.86	225.05 ± 32.86	159.09 ± 2.80	143.96 ± 23.49
M/F	46/63	120/189	16/8	30/55	54/32	66/157
Age (years)	29.50 ± 3.77	28.64 ± 3.90	29.54 ± 3.86	29.48 ± 3.77	28.23 ± 3.58	28.80 ± 4.01
***Race***						
White	73	248	19	54	78	170
Black	28	25	4	24	5	20
More than one	4	6	1	3	1	5
Unknown	4	2	-	4	-	2
Asian/Nat. Hawaiin	-	27	-	-	2	25
American Indian/Alaskan Nat.	-	1	-	-	-	1
Anxiety scores	4.29 ± 2.86	4.04 ± 2.80	4.75 ± 3.31	4.15 ± 2.71	4.34 ± 2.79	3.92 ± 2.80
Depression scores	4.64 ± 3.68	4.14 ± 3.65	5.08 ± 5.05	4.51 ± 3.18	4.57 ± 3.84	3.97 ± 3.56
Delay discounting; ddisc_AUC	0.33 ± 0.22*	0.41 ± 0.23*	0.30 ± 0.16^#^	0.35 ± 0.23^@^	0.38 ± 0.22	0.42 ± 0.23^#@^

Here, * denotes significant difference (*p* < 0.05) between the obesity and lean groups; # obesityHR and leanLR; @ obesityLR and leanLR.

*BMI* body mass index (kg/m^2^), *SD* standard deviation.

### Gambling task for fMRI

To measure decision-making, we used the HCP’s fMRI gambling task (GT) studies, developed by Delgado and colleagues [35], as it taps into the relevant cognitive systems [36]. The reward-related BOLD signal was measured during a card-guessing gambling task played for monetary reward, as previously described [37, 38]. Briefly, participants were required to guess the number (range 1–9) on the mystery card, which would determine if they win or lose money. The instructions were to press one of two buttons on the response box after guessing the number on the mystery i.e. if it is more or less than five. The participants were given feedback by revealing the card number they chose and a cue to inform them if they received a monetary reward, loss, or neutral (no reward/loss; for number 5) trial. The task was presented in blocks of eight trials that were either mostly reward (six reward trials pseudo-randomly interleaved with neutral and/or loss trials) or mostly loss (six loss trials interleaved with reward and/or loss trials). There were two mostly reward and two mostly loss blocks for each of the two runs, interleaved with four fixation blocks (15 s each). Although the participants gambled for potential monetary reward, all participants are rewarded with a standard amount of money during the task [37, 38].

### Delay discounting task

Immediate reward preference or devaluing of delayed rewards was assessed in the HCP dataset using an adjusting-amount monetary choice task. In this paradigm, each trial asks participants to indicate whether they would rather receive a smaller immediate reward (e.g., $100 today) or a larger delayed reward (e.g., $200 in 1 month). Briefly, participants were to make 5 choices of amounts based on the delayed amounts ($200 and $40,000) at each of six delay time points: 1 month, 6 months, 1 year, 3 years, 5 years, and 10 years. The delay choices based on both the delayed amounts ($200 and $40,000) were made in a certain fixed order of time combinations: (i) today vs. 6 months; (ii) today vs. 3 years; (iii) today vs. 1 month; (iv) today vs. 5 years; (v) today vs. 10 years; (vi) today vs. 1 year. The reward amounts were titrated based on participants’ choices until points of indifference (value for a “sixth” choice) were determined based on an increment or decrement from the immediate value of their fifth choice; that is, the point at which a person is equally likely to choose a smaller reward (e.g., $100) sooner versus a larger reward later (e.g., $200 in 3 years) [39]. The variable used to measure how steeply participants discounted delayed rewards was the area under the curve (AUC), a valid and reliable index of immediate reward preference [40]. In this study, we considered the average of the AUC variables for the $200 and $40,000 delayed reward conditions.

### fMRI data acquisition and preprocessing

Images for the HCP dataset were acquired using a customized Siemens Skyra 3-T scanner with a 32-channel Siemens receiver head coil and a body transmission coil. T1-weighted high-resolution structural images were acquired using a 3D MPRAGE sequence with 0.7 mm isotropic resolution (FOV = 224 × 224 mm, matrix = 320 × 320, 256 sagittal slices, TR = 2400 ms, TE = 2.14 ms, TI = 1000 ms, FA = 8°) and used to register functional MRI data to a standard brain space. fMRI data were collected using gradient-echo echo-planar imaging (EPI) with 2.0 mm isotropic resolution (FOV = 208 × 180 mm, matrix = 104 × 90, 72 slices, TR = 720 ms, TE = 33.1 ms, FA = 52°, multiband factor = 8253 frames, ~3 m, and 12 s/run) [38, 41].

Imaging data were analyzed with Statistical Parametric Mapping (SPM12, Welcome Department of Imaging Neuroscience, University College London, UK). Standard image preprocessing was performed. Images of each subject were first realigned (motion corrected). A mean functional image volume was constructed for each subject per run from the realigned image volumes. These mean images were co-registered with the high-resolution structural MPRAGE image and then segmented for normalization with affine registration followed by nonlinear transformation. The normalization parameters determined for the structural volume were then applied to the corresponding functional image volumes for each subject. Finally, the images were smoothed with a Gaussian kernel of 6 × 6 × 6 mm at full width at half maximum.

### Imaging data modeling

We modeled the BOLD signals to identify regional brain responses to win block versus neutral, loss block versus neutral, and win block versus loss. A statistical analytical block design was constructed for each subject, using a general linear model (GLM) with a boxcar each for win or loss blocks convolved with a canonical hemodynamic response function (HRF). Realignment parameters in all 6 dimensions were entered in the model as covariates. The GLM estimated the component of variance that each of the regressors could explain. In the first-level analysis, we constructed for individual subjects a statistical contrast of win block versus neutral, loss block versus neutral, and win block versus loss block to evaluate brain regions that responded to wins and losses and that responded differently to wins and losses. The contrast images (difference in β) of the first-level analysis were then used for the second-level group statistics.

As we observed that in our dataset the percentage of males was higher in obesityHR groups while the percentage of females was higher in leanLR groups, we assessed the effect of gender on brain activation during the gambling task by comparing males and females from the entire sample using a two-sample *t*-test. For group and sub-group analysis, we used a full-factorial general linear model with the independent, between-group factors of interest as BMI (groups: obesity and lean) and, alcohol drinking (groups: HR and LR) and four levels (obesityHR, obesityLR, leanHR, and leanLR), including age and sex as control covariates in SPM12. Multiple sub-group comparisons were made where we first used a standard double threshold method; first chose a cluster forming voxel threshold of *p* < 0.025 with *k* > 84 (minimum of 84 neighboring voxels), and then applied a threshold of *p* < 0.05 to correct for family-wise error (FWE) across the *p*-values of the surviving clusters [42]. Effectively, this combined voxel- and cluster-level statistic reflects the probability that a cluster of a given size, consisting only of voxels with *p* < 0.001, would occur by chance in data of the given smoothness. The surviving clusters were then used to form ROIs around the voxel with peak intensity in that cluster for further comparisons. The Marsbar tool in SPM12 was used to extract peak activation differences following significance thresholding and entered into an SPSS data matrix to assess the differential sub-group activations.

### Statistical analysis

We found < 1.0% missing data for all variables of interest. The participants with complete data were retained for further analysis. Levene’s test was applied to assess the equality of variances across the groups. Since we observed unequal variance in weight and BMI between the groups, we used Welch’s t-test to examine between-group differences in patient characteristics. The mean ranks of the ddisc_AUC measures between the groups were not equal. Mann–Whitney non-parametric tests were used to determine the differences in ddisc_AUC measures between the groups and sub-groups. Mann-Whitney was also used to determine the difference in number of drinks of alcohol between the subgroups. We conducted a two-way analysis of variance to investigate the effect of BMI and alcohol drinking on ddisc_AUC measures. To address potential confounders, we included age and sex as covariates (see Table 1). A threshold of *p* < 0.05 was considered for reporting data significance. All analyses were done using SPSS software.

## Results

In the obesity group, there were 24/109 (22%) individuals with high AUD risk and 85/109 (78%) with low AUD risk and in the lean group there were 86/309 (28%) individuals with high AUD risk and 223/309 (72%) with low AUD risk but differences in group composition were not significant (χ^2^ value = 1.1205; d*f* = 1; *p* = 0.29). The mean number of drinks consumed (intensity) was significantly higher in obesityHR (4.75 ± 1.11) compared to leanHR (4.02 ± 1.38; *p* = 0.03). However, the mean frequency of alcohol drinking did not differ between obesityHR (2.42 ± 1.18) and leanHR (2.23 ± 0.93; *p* = 0.06). Demographic details of subjects in each group are provided in Table 1.

### fMRI BOLD activations to win and loss

We examined regional responses to the win versus loss contrast in a full factorial model for sub-group comparisons. We observed the main effects of BMI during the win > loss contrast with significant cluster activations in the right postcentral gyrus (PoG), superior parietal lobule (SPL), and precentral gyrus (PrG) (Table 2). The main effects of alcohol drinking included clusters located in the left superior temporal gyrus (STG), middle temporal gyrus (MTG), and parietal operculum (PO) (Table 2). A positive interaction between BMI and alcohol drinking was observed for BOLD activations in clusters involving right PCu and left PrG, angular gyrus (AnG), supramarginal gyrus (SMG), and parietal operculum (PO) (Supplementary Fig. S2, Table 2).

**Table 2:** Groups showing significant brain region activations in response to wins versus losses.

Groups Region	Cluster size (k)	Peak voxel (Z)	Cluster FWE *P*-value	MNI coordinate		
				X	Y	Z
***The main effect of BMI***						
Postcentral gyrus_R	649	4.66	0.008	22	−40	72
Superior parietal lobule_R		3.59		14	−48	68
Precentral gyrus_R		3.32		40	−24	62
***The main effect of Alcohol Drinking***						
Superior temporal gyrus_L	854	3.94	0.001	−44	−34	2
Parietal operculum_L		3.71		−52	−24	16
Middle temporal gyrus_L		3.50		−50	−42	6
***BMI × Alcohol drinking***						
Postcentral gyrus_R	1224	3.91	0.000	32	−28	48
Precentral gyrus_L		3.66		−8	−34	68
Precuneus_R		3.74		6	−48	58
Angular gyrus_L	597	3.37	0.013	−50	−54	26
Supramarginal gyrus_L		3.04		−48	−44	42
Parietal Operculum_L		2.98		−60	−28	16
***ObesityHR*** ***>*** ***ObesityLR***						
Posterior insula_L	2340	4.05	0.000	−44	−18	−2
Superior temporal gyrus_L		3.93		−44	−34	2
Cuneus_L		3.67		−22	−72	24
Cerebellum_L	717	3.88	0.042	−8	−42	−26
Cerebellum_R		3.84		14	−42	−28
Cerebellar vermal lobules (VIII–X)		3.80		−4	−54	−30
Postcentral gyrus_R	1651	3.80	0.000	32	−28	48
Precuneus_R		3.66		4	−48	58
Posterior cingulate gyrus_R		3.61		8	−42	40
Middle cingulate gyrus_R	615	3.73	0.027			
Supplementary motor cortex_R		3.48				
***LeanHR*** ***<*** ***LeanLR***						
Medial segment precentral gyrus_R	741	3.27	0.035	6	−32	66
Superior frontal gyrus_L		3.14		−8	−22	70
Precentral gyrus_R		3.07		30	−18	64
***ObesityHR*** ***>*** ***LeanHR***						
Postcentral gyrus_R	2615	4.51	0.000	22	−40	72
Superior parietal lobule_L		3.93		−12	−48	64
Precentral gyrus_R		3.63		22	−28	72
Posterior insula_L	1515	3.67	0.000	−44	−18	−2
Superior temporal gyrus_L		3.52		−54	−32	8
Lingual gyrus_L		3.45		−32	−44	0
***ObesityHR*** ***>*** ***LeanLR***						
Cerebelllum_L	734	4.78	0.037	−4	−54	−30
Cerebellum_R		3.64		12	−42	−28
Superior temporal gyrus_L	1677	4.05	0.000	−48	−40	6
Posterior insula_L		3.78		−44	−18	−2
Middle temporal gyrus_L		3.72		−54	−32	8
Middle Cingulate gyrus_R	596	4.02	0.029	8	6	24
Caudate_R		3.85		6	−4	36
Thalamus_R		3.33		6	−6	24
Postcentral gyrus_R	1441	3.72	0.000	22	−40	72
Superior parietal lobule_R		3.41		24	−54	58
Supramarginal gyrus_R		3.13		54	−28	50
***ObesityLR*** ***<*** ***leanHR***						
Inferior occipital gyrus_L	1642	3.90	0.000	−24	−90	0
Lingual gyrus_L		3.82		−12	−90	−10
Calcarine cortex_L		3.74		−12	−90	0

Note: *LR* low-risk, *HR* high-risk, *L* left, *R* right. Coordinates refer to the cluster peak voxel in mm (MNI).

**Fig. 1:**
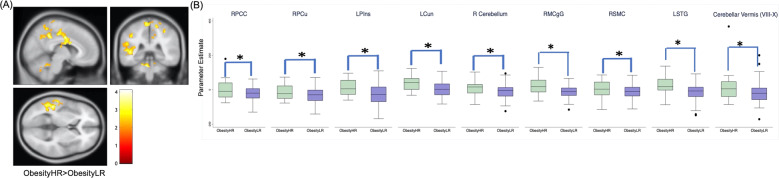
Shows the sub-group differences in regional responses on full factorial analysis of the contrast (win > loss) between obesityHR/obesityLR with age and sex as covariates. The corresponding BOLD image (**A**) shows the regional activation while, box plot (**B**) depicts the difference in extracted beta estimates from the activated clusters between the groups. The initial clustering threshold was chosen as *p* = 0.025, with *k* > 84; final *p*FWE < 0.000. All clusters with cluster *p* < 0.05 familywise error (FWE) of multiple comparisons are shown in Table 2. Here * signifies *p* < 0.05 between the groups.

**Fig. 2:**
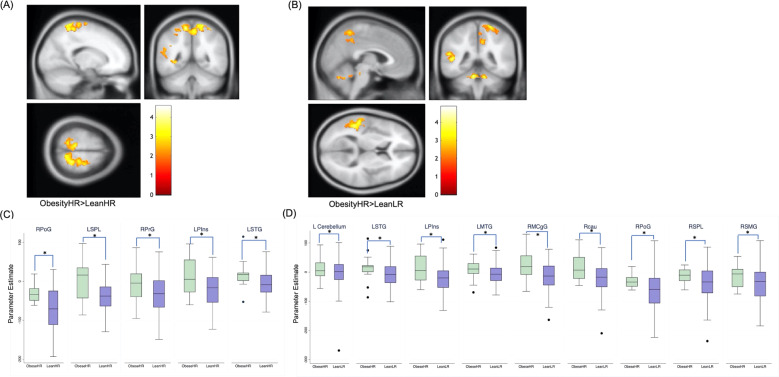
Shows the sub-group differences in regional responses on full factorial analysis of the contrast (win > loss) between obesityHR/leanHR and obesityHR/leanLR with age and sex as covariates. The corresponding BOLD images (**A**) & (**B**) show the regional activation while, box plots (**C**) & (**D**) depict the difference in extracted beta estimates from the activated clusters between the groups. The initial clustering threshold was chosen as *p* = 0.025, with *k* > 84; final *p*FWE < 0.000. All clusters with cluster *p* < 0.05 familywise error (FWE) of multiple comparisons are shown in Table 2. Here *p < 0.05 between the groups.

Analysis of activation differences between sub-groups for the win-loss contrast showed for obesityHR relative to obesityLR, greater activations in right PCC, PCu, middle cingulate gyrus (MCgG), the supplementary motor cortex (SMC), left STG, posterior insula (PIns), cuneus (Cu), bilateral cerebellum and cerebellar vermal lobules VIII–X (Fig. 1A&B). The obesityHR relative to leanHR comparison, revealed greater activation in clusters in right PoG, PrG, and left SPL, PIns, STG, and lingual gyrus (LiG) (Fig. 2A&C). The obesityHR relative to leanLR comparison revealed greater activation in right and left cerebellum, MCgG, Caudate (Cau), PoG, SPL, SMG, STG, PIns, and MTG (Fig. 2B&D). There were no differences between leanHR and obesityHR groups.

**Fig. 3:**
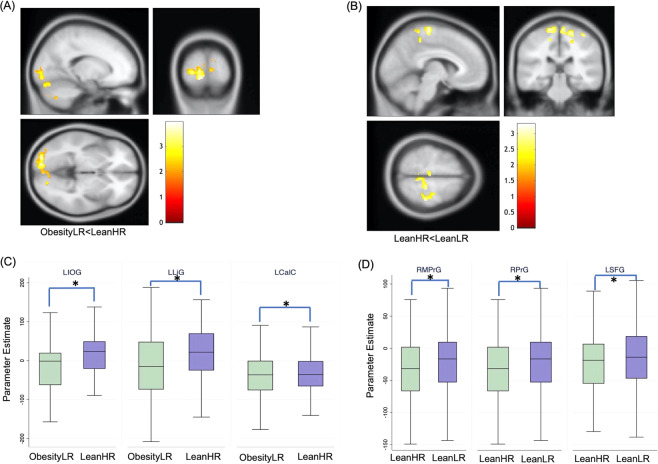
Shows the sub-group differences in regional responses on full factorial analysis of the contrast (win > loss) between leanHR/obesityLR and leanHR/leanLR with age and sex as covariates. The corresponding BOLD images (**A**) & (**B**) show the regional activation while, box plots (**C**) & (**D**) depict the difference in extracted beta estimates from the groups’ activated clusters. The initial clustering threshold was chosen as *p* = 0.025, with *k* > 84; final *p*FWE < 0.000. All clusters with cluster *p* < 0.05 familywise error (FWE) of multiple comparisons are shown in Table 2. Here *p < 0.05 between the groups.

The leanHR relative to obesityLR comparison showed greater activation in the left inferior occipital gyrus (IOG), LiG, and calcarine cortex (CalC) (Fig. 3A&C). The leanLR relative to leanHR showed greater activation in right PrG, medial PrG (mPrG), and left superior frontal gyrus (SFG) (Fig. 3B&D). The locations of the subgroup comparison are summarized in Supplementary Fig. S3 and details for the cluster in Table 2.

Gender comparison revealed significantly higher frontal activation (bilateral SFG, and left MFG, MPrG, MPoG) in males compared to females in the whole dataset in the win>loss contrast (see Supplementary Fig. S4). There were no regions where females showed greater activation than males. We did not observe significant associations between anxiety and depression scores and brain activation signals for any of the sub-groups.

### Delay discounting behavioral measures

We observed significantly lower ddisc_AUC values in the obesity and HR group as compared to the lean and LR group (*p* = 0.001; 0.04), indicating greater discounting of delayed rewards (i.e., greater tendency to choose smaller rewards now, as opposed to larger rewards later) (Supplementary Fig. S5; Table 1). Differences in delay discounting were also observed between sub-groups, where obesityHR showed significantly lower (*p* = 0.001) values compared to leanLR similarly, obesityLR had lower values than leanLR groups (*p* = 0.01) (Supplementary Fig. S5; Table 1). Further, a two-way analysis of variance showed significant main effects for both BMI [*F*(1,417) = 26.36; *p* = 0.04] and alcohol drinking [*F*(1,417) = 10.05; *p* = 0.04] on ddisc_AUC measures. However, we did not see an interaction effect of BMI*alcohol drinking [*F*(1,417) = 0.04; *p* = 0.85].

## Discussion

In this study, 22% of obesity and 28% of lean subjects were at high-risk of AUD and while drinking intensity was significantly higher in obesityHR compared to leanHR their frequency of consumption did not differ. High-intensity drinkers (regardless of frequency) reportedly have higher BMI, which is most likely associated with their increased intake of calories from foods and drinks [43, 44]. The main dietary macronutrients that serve as sources of energy are fat (38 kJ/g), carbohydrates and protein (each 17 kJ/g), and to lesser extent alcohol (ethanol) (29 kJ/g). Alcohol is more energy-dense than carbohydrates and proteins, and calories from consumed alcohol are additive to that from other dietary sources, which can result in a positive energy balance and weight gain [45]. However, in the HCP data we cannot determine if and how much calories from drinking contributed to an individual’s weight since it does not provide sufficient details on daily calorie food and alcohol consumption and physical activity.

Our fMRI results showed an interaction between BMI and alcohol drinking in PCu and PrG, which are part of the default mode network (DMN) and implicated in self-related processing, memory, and salience attribution [46–48]. The PrG is typically deactivated during task-based activation and is anti-correlated with brain networks associated with executive functioning [49–51]. The angular gyrus was also associated with BMI and alcohol drinking. The AG is a part of the inferior parietal lobule that mediates automatic “bottom-up” attentional resources, and its increased activation is strongly related to high memory performance [52]. The activation of angular and parietal regions in the left hemisphere observed most likely reflects the processing of memory and uncertainty components encountered during the gambling task [53].

We observed heightened activation of DMN (Cu/PCu, PCC), the primary motor cortex (SMC, MCgG) and of regions that aid in decision-making during uncertain choices (PIns), regions implicated in attentional deficits (Cerebellar vermal lobules VIII–X), and negative perception (STG) in participants with high BMI and high-risk for AUD relative to their low-risk counter group. Recently, greater BOLD activation in DMN regions, including the ventromedial prefrontal cortex (vmPFC), PCC, and right PrG was reported in subjects with obesity while performing the N-back task [54]. Similarly, DMN regions (PCC and precuneus) were shown to have greater activation during drug‐cue exposure in cocaine [55], alcohol [56], nicotine [57–60], and cannabis use disorders [61–63]. Thus in line with this reasoning, we interpret the activation pattern in the high-risk groups to reflect their inability to maintain attention and focus, which in turn may facilitate impulsive choices. Furthermore, the greater activity observed in the parietal lobule and cerebellum might pertain to higher uncertainty associated with choices, which results in negative perception about the outcome and hence loss during the task [64]. The increased STG activity in obesityHR individuals is understandable as the loss involved in the task elicits negative emotions [65]. Therefore, the neural activation pattern in obesityHR group during the gambling task corroborates findings from previous studies on decision-making deficits in obesity [66–69] and AUD [67, 70, 71] who prefer short-term disadvantageous rewards (despite negative long-term consequences) over advantageous long-term ones.

Increased activation in DMN and in regions implicated in uncertain decisions in obesityHR as compared to leanHR and leanLR groups is consistent with prior findings of increased DMN activation in obesity compared to lean individuals, which was interpreted to reflect increased attention to internal states like appetite or gut signals [72]. The common themes here relate to deficits in attention, memory, and increased uncertainty attributed to the mental processes that underlie decision-making. Since subjects with both high BMI and chronic alcohol consumption recruited brain regions associated with enhanced sensitivity to reward in the gambling task compared to lean BMI groups both at high and low-risk of AUD (HR and LR), we carried out further between-group comparisons with an aim to explore and understand if this is an effect of high BMI or excessive alcohol consumption or a combined effect of these addictive drives. We observed that the individuals who were lean and at a high-risk of AUD in comparison to the obesityLR group recruited occipital regions related to increased visual attention. There has been growing debate on the nonlinear effect of alcohol drinking frequency on BMI [73]. Moreover, alcohol and carbohydrates might compete for the same neuronal receptors leading to the suppressed intake of one nutrient for the intake of the other [74]. Alcohol drinking frequency was similar in obesityHR (4–7days/week; 24%, 1–3 days/week; 76%) and leanHR (4–7days/week; 21.3%, 1–3 days/week; 78.7%) groups. Thus the BOLD activation differences in these groups, suggest that BMI is an independent variable in the neural processing of the gambling task by these groups of individuals.

Further, we also observed increased activation in the motor [mPrG, PrG, and PFC (SFG)] regions of leanLR individuals compared to leanHR. PFC is mainly concerned with executive control, and metabolic activity in this region has been demonstrated to negatively correlate with BMI and alcoholism [75, 76]. PFC also has a critical role in controlling/inhibiting negative impulsive behavior [77]. Dopamine plays a significant role in cost-benefit decision-making preferences [78]. Chronic alcohol intake is associated with pronounced alterations in dopaminergic neurotransmission [79], consequently compromising the function of the PFC, which receives these dopaminergic inputs. Similarly, obesity has been associated with reduced dopaminergic signaling and impaired PFC activity [80]. Thus for the leanHR participants alcohol use might have resulted in worse executive and inhibition control than in the leanLR individuals. Though a priori we would have expected that impairments in PFC would have been even more severe in obesityHR than in leanHR this was not the case. Instead, obesityHR compared to leanHR had greater activation in sensory regions whereas there were no regions for which leanHR had greater activation than obesityHR.

We also compared the delayed discounting task measures between these groups, which complements the gambling task by assessing preference for small immediate rewards versus large delayed rewards, another key component of decision-making. In agreement with prior findings, we observed significant behavioral differences between obesity and lean groups. The obesity group showed stronger discounting of future monetary rewards than the lean group. This may relate to the preference of obesity individuals for highly rewarding unhealthy foods despite their long-term detrimental effects as compared to lean individuals. We also observed that the delayed discounting measure differed in HR and LR both in obesity and lean individuals. Although these differences were also apparent between the sub-groups with obesityHR and obesityLR having lower measures compared to the leanLR group, the interaction between BMI and alcohol drinking was not significant. The delayed discounting measure did not distinguish between obesity and high alcohol drinking levels, which as for the gambling task fMRI results, suggests that obesity and chronic alcohol drinking have independent negative effects on decision-making.

There are certain limitations to the present study. Firstly, the HCP data lacks a measure of reward anticipation, which is another key dimension of decision-making behavior. Secondly, we used only the gambling task-fMRI. Functional connectivity studies using rs-fMRI might provide better information on how intrinsic network function supports decision-making behavior. Thirdly, this explorative study, which solely relies on BMI as a measure of obesity, needs to be extended with precise adiposity measures, other anthropometrics, or metabolic functioning. Moreover, a more detailed analysis of the type of alcohol consumed would give more insights into these findings considering the differential impact of alcohol types on weight changes reported across studies. The fourth limitation is that participants were predominantly of European ancestry and individuals from other ethnicities may carry a higher risk of obesity and have a higher burden for its deleterious consequences [81]. Thus the limited ethnic breakdown of participants in the HCP dataset limits the generalizability of our results. Finally, our subgroups differed in sex composition, with a higher percentage of males in the high-risk AUD groups relative to other groups. While we controlled for sex (as well as age) we can not completely rule out potential sex differences in activation responses and distinct interaction between sex, BMI, and alcohol drinking, which should be investigated in future studies with larger samples.

## Conclusion

The current study documents differences in the neural activation patterns during the gambling task in obesity and lean participants at high and low-risk of AUD. The findings demonstrate a significant impact of BMI and alcohol consumption, and interaction of the two, on interoceptive regions including posterior DMN and parietal operculum during the gambling task. However, we found significant heterogeneity in the discounting measures within and across groups. Moreover, delay discounting was seen to independently predict BMI and alcohol drinking. Together, these findings highlight distinct associations of obesity and high-risk alcohol drinking with two key constituents of decision-making behavior.

## Supplementary information

Supplementary Figure legends

Supplementary Fig S1

Supplementary Fig S2

Supplementary Fig S3

Supplementary Fig S4

Supplementary Fig S5
