# Supplementary material for: Relationship between BMI and alcohol consumption levels in decision making
Source: Int J Obes (Lond). 2021 Aug 6;45(11):2455–63. doi: 10.1038/s41366-021-00919-x (PMC8528710; doi:10.1038/s41366-021-00919-x)
Supplement: Supplementary file 1 — Supplementary Figure legends [file 41366_2021_919_MOESM1_ESM.docx]

**Supplementary Fig. S1.** Diagram depicting a detailed workflow of subjects selection criteria.

**Supplementary Fig. S2.** BOLD activation in brain regions during the win>loss contrast of the gambling task showing main BMI effect (A); main alcohol drinking effect (B); and a significant positive interaction between BMI and alcohol drinking (C). Initial clustering threshold was chosen as p = 0.025, with k > 84; final pFWE < 0.003.

**Supplementary Fig. S3.** Schematic representation of the human brain showing regions activated in response to reward sensitivity in (i) obesity>lean, (ii) HR>LR AUD and, (iii) BMI*alcohol drinking interaction, (iv) obesityHR>obesityLR, (v) obesityHR>leanHR/LR, (vi) obesityLR>leanHR, (vii) leanHR>obesityLR, (viii) leanLR>leanHR.

Note: HR = high risk; LR = low risk; AUD = alcohol use disorder; SFG = superior frontal gyrus; PrG = precentral gyrus; PoG = postcentral gyrus; SMC = supplementary motor cortex; AnG = angular gyrus; SPL = superior parietal lobule; SMG = supramarginal gyrus; PCC = posterior cingulate cortex; MCgG = middle cingulate gyrus; Cau = caudate; PCu = precuneus; Cun = cuneus; CalC = cal carine cortex; IOG = inferior occipital gyrus; LiG = lingual gyrus; PO = parietal operculum; PIns = posterior insula; STG = superior temporal gyrus; MTG = middle temporal gyrus.

**Supplementary Fig. S4.** BOLD activation in brain regions during the win>loss contrast of the gambling task between male>female (A). The corresponding box plot (B) depicts the difference in extracted beta estimates from the activated clusters between the groups. Initial clustering threshold was chosen as p = 0.025, with k > 84; final pFWE < 0.000. All clusters with cluster p < 0.05 familywise error (FWE) of multiple comparisons. Here * signifies p<0.05 between the groups.

**Supplementary Fig. S5.** Box plot representation of the difference in delayed discounting measure between obesity and lean individuals (A) as well as those at high and low risk of AUD (B). While plot (C) and (D) illustrates the differences in delayed discounting measures between obesityHR/LeanLR and ObesityLR/LeanLR. Here * signifies p<0.05 between the groups.
