# Supplementary figures and images for: Relationship between BMI and alcohol consumption levels in decision making
Source: Int J Obes (Lond). 2021 Aug 6;45(11):2455–63. doi: 10.1038/s41366-021-00919-x (PMC8528710; doi:10.1038/s41366-021-00919-x)

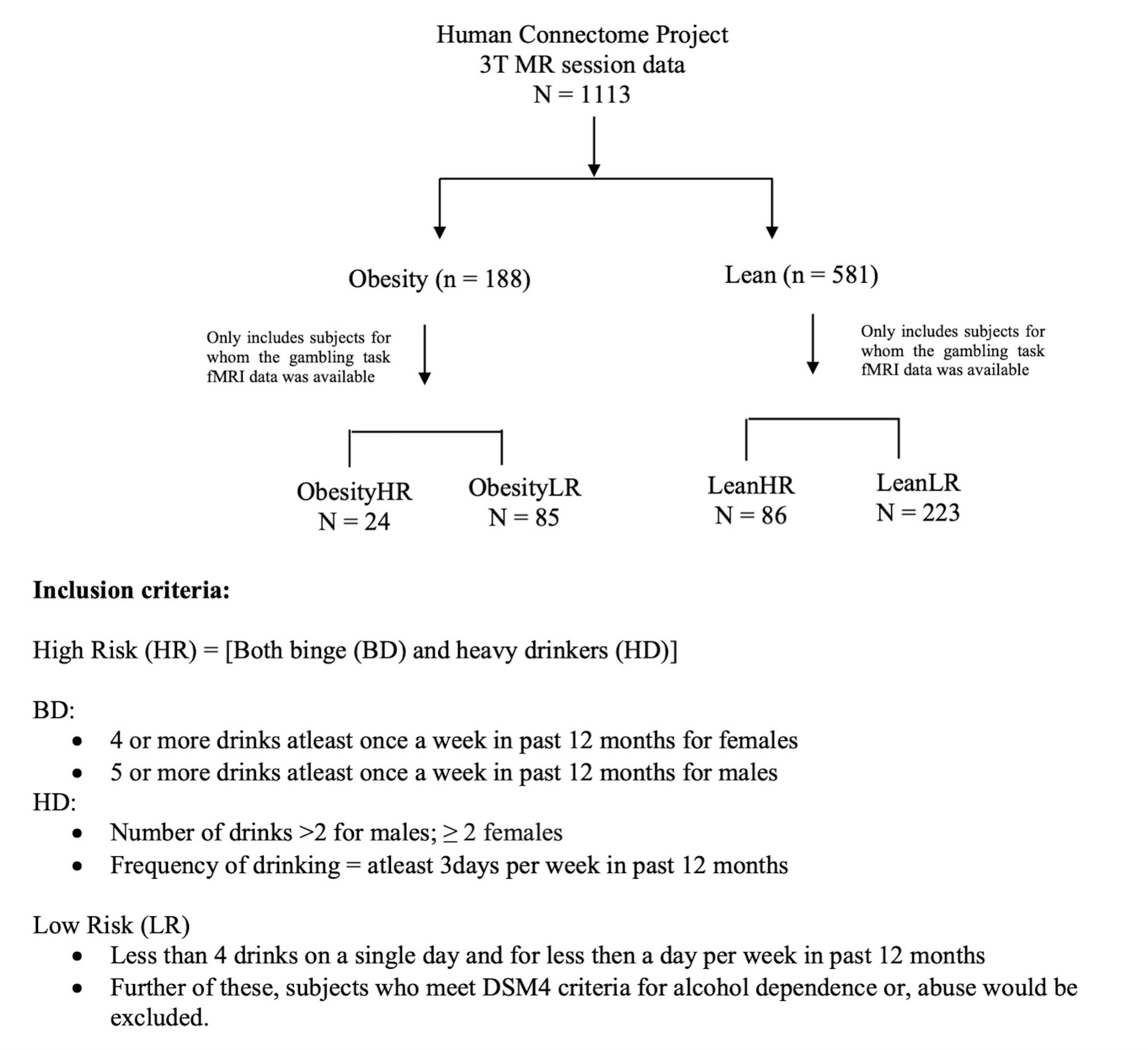

Supplement: Supplementary file 2 — Supplementary Fig S1 [file 41366_2021_919_MOESM2_ESM.jpg]

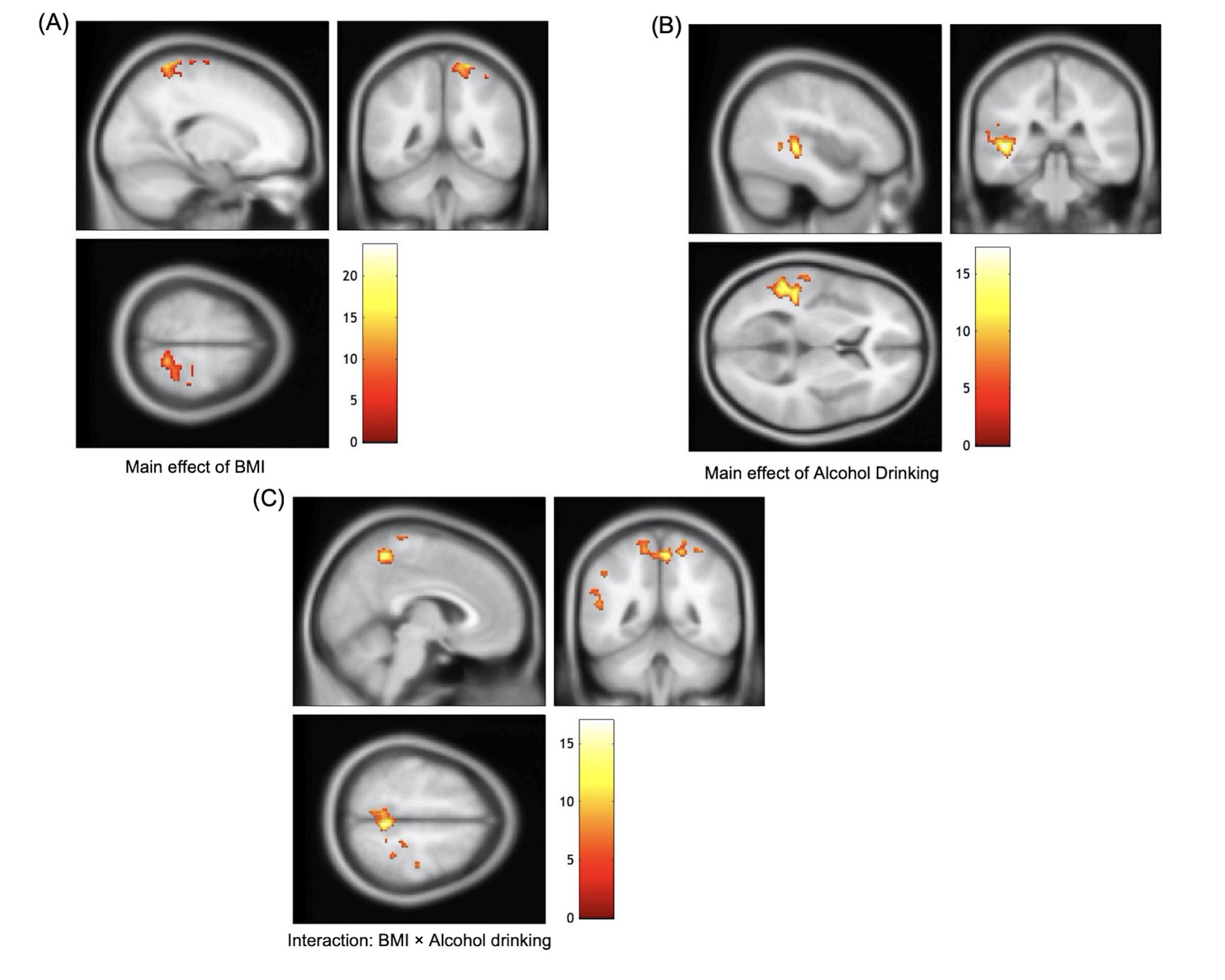

Supplement: Supplementary file 3 — Supplementary Fig S2 [file 41366_2021_919_MOESM3_ESM.jpg]

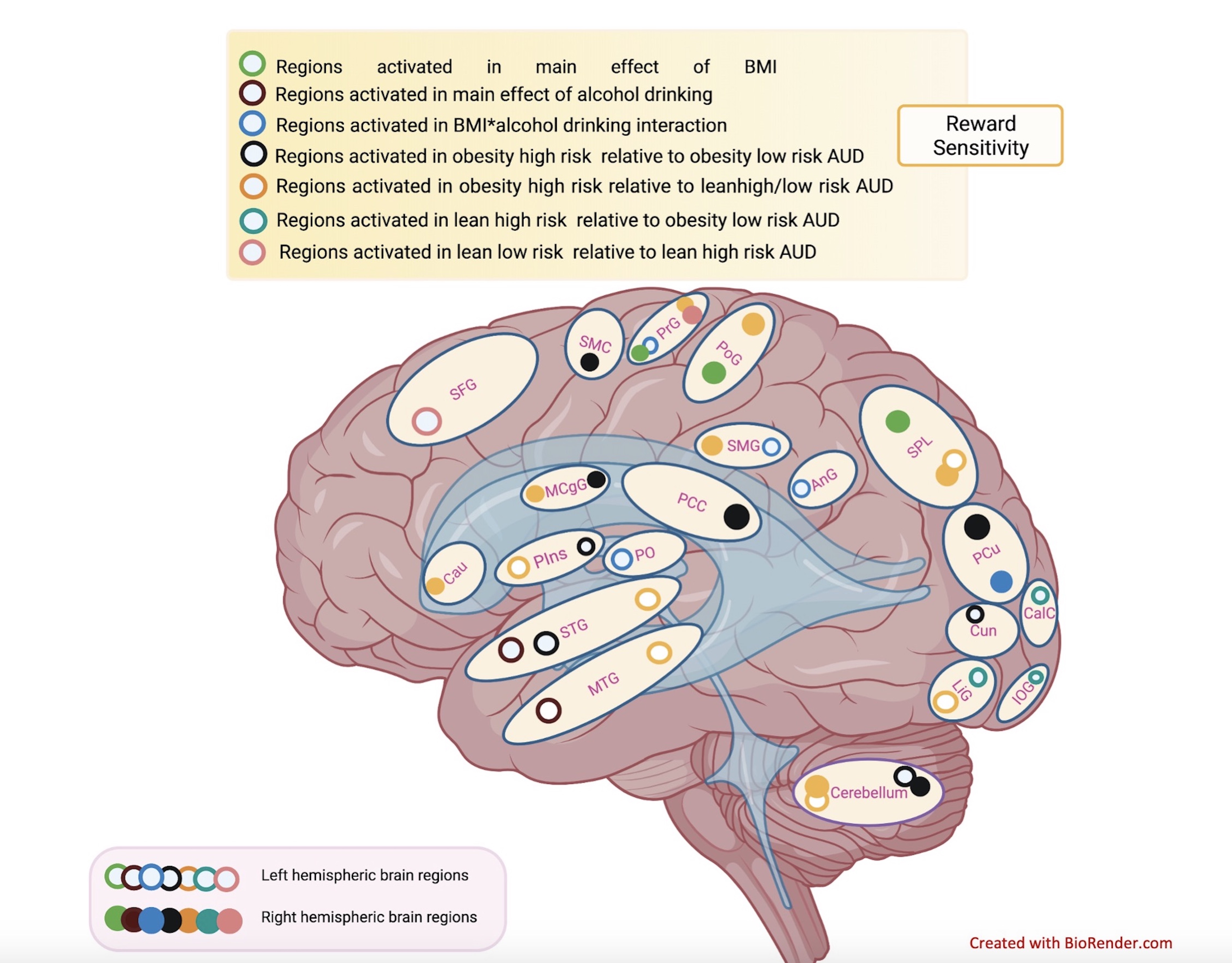

Supplement: Supplementary file 4 — Supplementary Fig S3 [file 41366_2021_919_MOESM4_ESM.jpg]

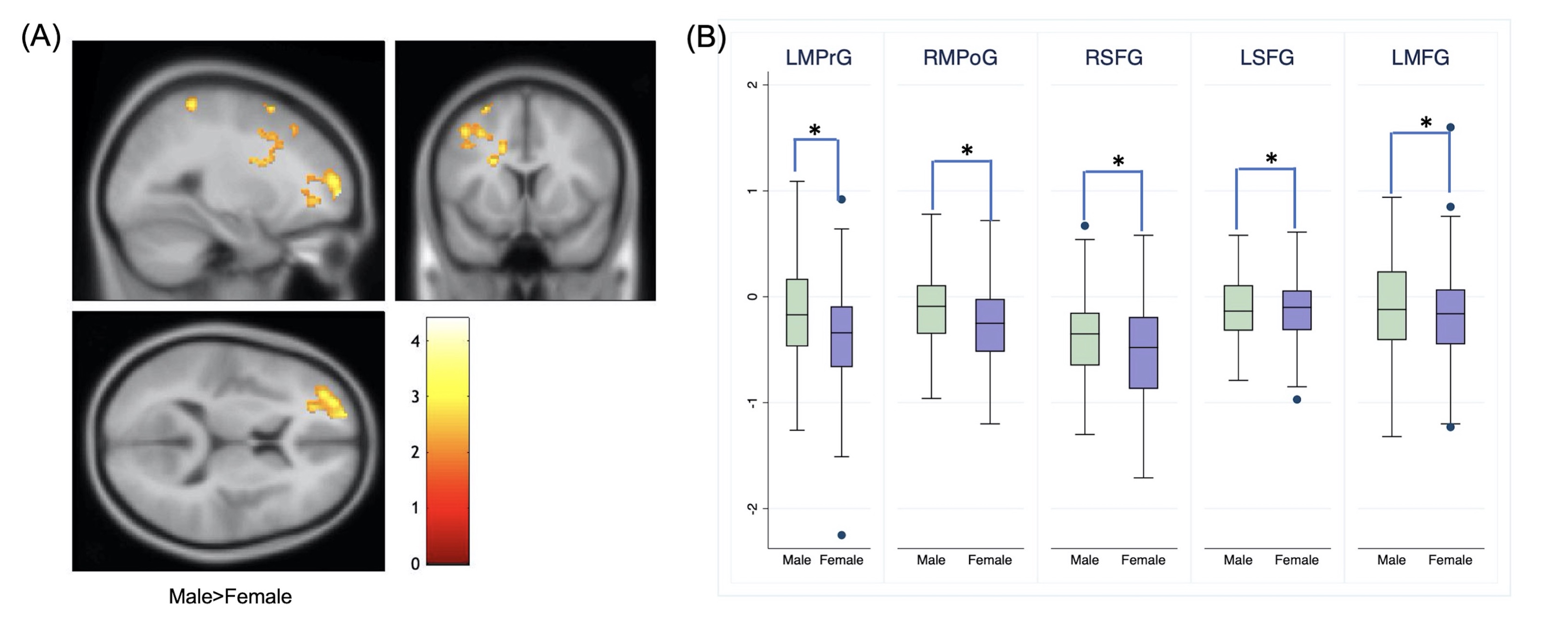

Supplement: Supplementary file 5 — Supplementary Fig S4 [file 41366_2021_919_MOESM5_ESM.jpg]

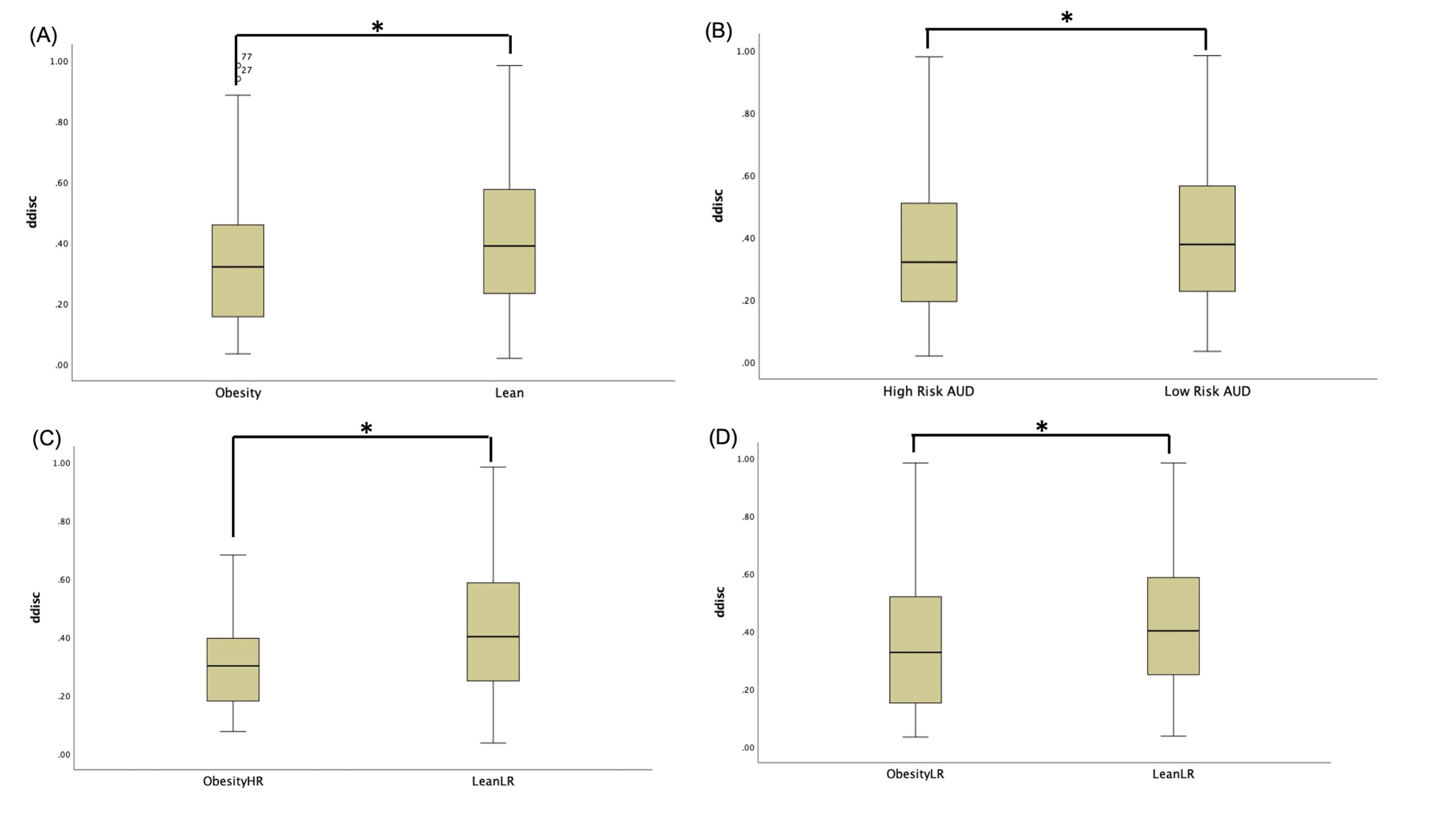

Supplement: Supplementary file 6 — Supplementary Fig S5 [file 41366_2021_919_MOESM6_ESM.jpg]
